# Author Correction: Self-supervised predictive learning accounts for cortical layer-specificity

**DOI:** 10.1038/s41467-025-65076-5

**Published:** 2025-10-22

**Authors:** Kevin Kermani Nejad, Paul Anastasiades, Loreen Hertäg, Rui Ponte Costa

**Affiliations:** 1 Centre for Neural Circuits and Behaviour, Department of Physiology, Anatomy and Genetics, University of Oxford, Oxford, United Kingdom; 2 Bristol Computational Neuroscience Unit, Intelligent Systems Lab, Faculty of Engineering, University of Bristol, Bristol, BS8 1TH United Kingdom; 3 Department of Translational Health Sciences, University of Bristol, Whitson Street, Bristol, BS1 3NY United Kingdom; 4 Technische Universität Berlin & Bernstein Center for Computational Neuroscience Berlin, 10115 Berlin, Germany

**Keywords:** Learning algorithms, Network models, Cortex

Correction to: *Nature Communications* 10.1038/s41467-025-61399-5, published online 4 July 2025

In the version of the article initially published, Fig. 2a was an earlier, incorrect version, and the image and legend have been updated. For comparision, the original figure is available as Supplementary Information accompanying this amendment. Additionally, in eq. (5), the lower bracket, above “prediction, ${\hat{z}}_{t}^{L5}$”, previously encompassed the entire upper parenthetical expression. The equation has been amended as shown below:

$${{\mathcal{C}}}_{{{{{L}}}2/3} \rightarrow {{{{L}}}5}}=\frac{1}{2} {(\underbrace{W_{{{{{L}}}2/3} \rightarrow {{{{L}}}5}} \cdot {{{z}}}^{{{{L}}}2/3}_t}_{{{\rm{prediction}}}, {\hat{{{z}}}}^{{{{L}}}5}_t} - {{{z}}}^{{{{L}}}5}_t )}^{2}$$

The figure, caption and equation are updated in the HTML and PDF versions of the article.

## Supplementary information

Original, uncorrected Fig. 2

