# Supplementary material for: Self-supervised predictive learning accounts for cortical layer-specificity
Source: Nat Commun. 2025 Jul 4;16:6178. doi: 10.1038/s41467-025-61399-5 (PMC12227776; doi:10.1038/s41467-025-61399-5)
Supplement: Supplementary file 1 — Supplementary Information [file 41467_2025_61399_MOESM1_ESM.pdf]

Supplementary Information

Numerical details

We model each layer of the neocortical microcircuit as a linear transformation of its input followed by a non-linearity. The network layers are specified with the following default neuron counts: Layer 4 (L4) and Layer 2/3 (L2/3) each have 128 neurons, and Layer 5 (L5) has 16 neurons. In some experiments, we varied the number of neurons in each layer to explore the effect of neuronal density on the outcomes being evaluated. For experiments aligning with those conducted by Jordan and Keller [1], we adjusted the neuron counts in L2/3 and L5 to 32 and 16, respectively, to more closely match the proportion of neurons recorded from each layer during their experiments.

Network parameters are optimised using the standard ADAM optimiser with a learning rate of 0.001, and beta parameters ($\beta_1 = 0.9$, $\beta_2 = 0.999$; see below for results with Stochastic Gradient Descent). To ensure efficient learning, we employ standard stochastic gradient descent with a batch size of 32 and continue training until convergence, typically around 1000 epochs.

ANN parameters are initialized using a uniform distribution $\mathcal{U}(-\sqrt{k}, \sqrt{k})$, where k represents the number of neurons per layer. We use backpropagation for network training, except in experiments shown in Figure 6, where we explore alternative ways of setting the feedback weights. All results reflect an average across 5 random seeds.

Experiments were performed on the BluePebble supercomputer (University of Bristol), primarily using GeForce RTX 2080 Ti GPUs, with occasional CPU usage.

L23-to-L5 feedback experiments

This experiment investigates the importance of the feedback connection from L5 to L2/3 in model performance. Instead of using the optimal feedback weight matrix ($W_{\text{L5}\rightarrow\text{L2/3}} = W^{T}_{\text{L2/3}\rightarrow\text{L5}}$), as derived using the backpropagation algorithm, we replace it with random weights in line with a variant of backpropagation known as feedback alignment [2]. In addition, we introduce a probability variable (P) to control the density of the feedback connections ($W_{\text{L5}\rightarrow\text{L2/3}}$) compared to the forward connections ($W_{\text{L2/3}\rightarrow\text{L5}}$). Each feedback connection has a probability $1 - P$ of being set to zero. For the results shown in Fig. 4 and S7 we created multiple model variants, each with varying feedback connection densities determined by this probability. All model variants are then trained on the sequential Gabor task.

Additional experiments

The role of L5 regularisation and L2/3 temporal self-supervised learning

Here, we investigate the importance of the L5 reconstruction cost on the learning capabilities of the model. We first demonstrate that removing the L5 reconstruction loss, which serves as a regulariser, leads to representational collapse. In this scenario, both L2/3 and L5 converge to a degenerate solution, producing near-identical results regardless of the input (Fig. S5a). Next, we explore alternative regularisation techniques, focusing on variance maximization as proposed in VICReg [3]. This method, shown to be analogous to Hebbian plasticity rules [4], successfully prevents representational collapse. With variance maximization as the L5 objective, L2/3 effectively learns to predict its input, while L5 develops distinct representations of the current input (Fig.S5b).

Finally, we examine the impact of modifying the L2/3 learning objective. Instead of the temporal self-supervised prediction task, we replace it with a simple regression task: predicting the speed associated with the current input. This modification significantly impacts L2/3's predictive ability. While L5 still learns to represent the input patterns, L2/3 loses its ability to predict its input, indicating a reduced capacity to capture the temporal structure of the data (Fig. S5c). These results underscore the importance of both the L5 regularisation strategy and the choice of the L2/3 objective function for achieving successful learning and representation in our model.

The influence of optimiser and regularisation on learning and sparsity

To investigate the robustness of our findings on neural response sparsity, we explored the impact of different optimiser and regularisation choices. First, we replaced the Adam optimiser with stochastic gradient descent (SGD) while retaining the reconstruction loss for L5. In this configuration, the model failed to learn, likely becoming trapped in local minima due to the shallow architecture and the limitations of SGD in navigating flat loss landscapes (Fig. S10a). However, when variance maximization was employed as the L5 regulariser, both SGD and Adam optimisers yielded qualitatively similar sparsity patterns across cortical layers (Fig. S10b). These patterns were consistent with our previous results in Figure 6, indicating that the observed sparsity trends are robust to variations in the optimiser as long

as a suitable regularisation strategy is employed for L5. Furthermore, we investigated the impact of learning rates on population sparseness, exploring a range of values: [0.005, 0.001, 0.0005, 0.0001]; we found that the Adam optimiser exhibited instability with larger learning rates, such as 0.01.

Effect of visuomotor coupling on neural responses

We also investigated whether our model could also replicate the distinct neural responses to visuomotor mismatch observed in coupled training (CT) and non-coupled training (NT) paradigms, as reported by Attinger et al. [5]. In these experiments, mice trained under CT conditions exhibited robust neural responses specifically to mismatch events, where visual flow was halted while locomotion persisted. Conversely, mice trained under NT conditions, where visual flow was independent of their movement, displayed responses to both mismatch and playback halt events in which a previously experienced visual flow was randomly initiated (Fig. S14a). Our model captures the fact that neurons in the CT condition respond to mismatch but not to playback halt, while neurons in the NT condition respond to both mismatch and playback (Fig. S14). This alignment with experimental data supports the validity of our model in capturing key aspects of visuomotor coupling effects on neural responses.

To simulate these paradigms, we modelled the L2/3 layer as a recurrent neural network (RNN) as follows:

$$\mathbf{z}_{t+1}^{\mathrm{L2/3}} = \sigma\left(W_{\mathrm{rec}}^{\mathrm{L2/3}} \cdot \mathbf{z}_t^{\mathrm{L2/3}} + W_{\mathrm{L4}\rightarrow\mathrm{L2/3}} \cdot \mathbf{z}_t^{\mathrm{L4}} + W_{\mathrm{td}\rightarrow\mathrm{L2/3}} \cdot \mathbf{I}_t^{\mathrm{td}}\right) \tag{1}$$

where W_{rec} is the recurrent weight matrix, and The term $W_{\mathrm{rec}}^{\mathrm{L2/3}} \cdot \mathbf{z}_t^{\mathrm{L2/3}}$ allows the layer to integrate information over previous timesteps. Note that for this model we do require Backpropagation Through Time (BPTT) to train the model.

This modification allowed the model to integrate information on a longer time scale, better capturing the temporal dynamics of visuomotor integration. Following training in both CT and NT conditions, we introduced mismatch and playback halt perturbations, as illustrated in Fig. S14a. Our model successfully replicated the key experimental findings: in the CT condition, L2/3 neurons exhibited responses to mismatch but not during playback halt, while in the NT condition, responses were observed under both conditions (Fig. S14b-e). This result highlights the ability of our model to capture the nuanced influence of visuomotor coupling on neural response properties. Interestingly, while our model replicates the distinct response patterns to mismatch and playback halt, the overall magnitude of mismatch responses is similar between CT and NT conditions, unlike in the original study[5]. This discrepancy could stem from factors not incorporated in our model, such as the modulation of error signals or neural gain during locomotion, potentially due to heightened arousal or attention. Investigating the role of such factors, perhaps through the incorporation of reinforcement learning mechanisms, constitutes a promising direction for future research that could further elucidate the mechanisms underlying the observed differences in mismatch response magnitudes between CT and NT conditions.

Visual Flow Responses inversely relate to mismatch responses in L2/3 and L5

To investigate whether our model exhibits the same inverse relationship between visual flow responses and mismatch responses observed experimentally, we simulated an open-loop paradigm where visual flow was randomly initialized to a high value after a period of low visual flow. This manipulation, mirroring experimental designs[1], allowed us to examine mismatch responses to the onset of visual flow, contrasting with the halt paradigm where mismatch responses were evoked by the halting of the visual flow. Consistent with experimental findings, our model demonstrated a reversal of mismatch response polarity in both L2/3 and L5. Neurons exhibiting positive mismatch responses during visual flow halt now displayed negative responses upon flow onset, and vice-versa (Fig. S13).

Self-supervision cost in L2/3

This control experiment examines the impact of a temporal self-supervised learning objective directly implemented in L2/3 and the resulting model's ability to learn sequential tasks. The model architecture, illustrated in panel a, features delayed input to L2/3 from L4, and an SSL loss encouraging L2/3 to predict the subsequent input in pixel space. Our results confirm that L2/3 successfully learns to anticipate the next input while L5 learns useful features of the input (Fig.S11b). However, this model does not reproduce key experimental findings by Jordan and Keller [1] concerning L5 responses to mismatches in stimulus sequences (Fig.S11c). Specifically, L5 in the model did not exhibit suppression of activity observed in biological L5 neurons during mismatch periods. This discrepancy suggests that while input prediction in pixel space can facilitate temporal learning, it may not fully capture the underlying neural mechanisms

of perceptual processing. This is also supported by the findings of Balestriero and LeCun[6] on the limitations of input reconstruction for perceptual learning.

Extended discussion

Neural encoding of error signals

One difference between our study and that of Jordan and Keller[1] is that our model simulates mismatch errors by directly computing one or two steps of error derivatives (backpropagation) with respect to L2/3 or L5 neurons (see Methods). In contrast, their (experimental) study uses membrane voltage as an indicator of mismatch between ascending sensory input and descending top-down information. Reconciling these two views in a biologically plausible manner remains an open question. However, there are two potential approaches by which our work can be extended so as to model both inference and learning through subthreshold activity (membrane voltage).

The first approach involves building on multiplexing theories of the backpropagation algorithm[7–9]. Following the multiplexing framework, the error signals originating in layer 5 (L5) could be kept separate from the inference signals. These error-like events, potentially in the form of bursts, would then propagate from L5 to L2/3, representing prediction errors in neuronal activity. Interestingly, in this first approach, it has recently been proposed that one way by which the brain can implement a biologically plausible version of the error backpropagation algorithm is by incorporating dendritic subcomponents that integrate top-down excitatory input with inhibitory inhibitory input from interneurons with somatostatin (SOM)[7,10]. When there is a mismatch between visual flow and motor/contextual input, inhibition from SOM interneurons in L2/3 has been observed to be reduced, leading to membrane depolarization in vivo[5] which can be interpreted as an error signal in backprop-like models[7,10]. This aligns with previous studies showing that increased activity in L2/3 pyramidal neurons, as occurs in response to a mismatch error, can drive a corresponding hyperpolarization in a subset of L5 pyramidal neurons[11,12]. Our current model does not incorporate subcellular compartments or different cell types; however, it will be of interest to expand upon these ideas in the future and incorporate these additional biological features into our model.

The second approach for combining neural dynamics with learning within the same framework would be to recast our model within a predictive coding framework[13,14]. Predictive coding jointly optimises both model parameters and neuronal activities, which could naturally lead to prediction errors observable in the activity of both L2/3 and L5 neurons. Note that these two views are not mutually exclusive, as has been previously demonstrated[14].

Timescales

The model we propose in this study operates over a narrow time horizon which is dictated by the delay associated with mono-synaptic communication across layers, which ranges from a few milliseconds up to 10ms depending on study[15–18]. However, sensory responses in the neocortex are often extended by tens or hundreds of milliseconds via recurrent interactions within and between cortical layers[19]. The primary visual thalamus itself possesses late activity independent of retinal input that extends for hundreds of milliseconds[20]. We show that our model can be made compatible with such recurrent network architectures (Fig. S14). Although spike-time-dependent plasticity operates in the range of milliseconds to tens of milliseconds[21], dendritic plateau potentials last much longer[22] and could allow plasticity across wider, behaviorally relevant temporal horizons (e.g.[23]).

Timescales of cortical network operations are also known to vary along the cortical hierarchy, with higher-order cortical regions operating over longer timescales than primary sensory areas[24,25]. Despite many similarities in the organisation of cortical networks across brain regions, there are also differences in gene expression, neuronal morphology, synapse density, receptor composition, and the relative ratio of certain types of inhibitory interneurons which are thought to influence the properties of the network[24,26]. Indeed, computational models of recurrent networks containing both excitatory and inhibitory units generate multiple timescales based on differences in network architecture[27]. This is consistent with the idea that the canonical cortical circuit represents a generic blueprint upon which region-specific adaptations can be overlaid[28].

Extrapolating across the cortical hierarchy

How, then, might our model operate across the cortical hierarchy? Here, we focus on sensory information, relayed from the primary sensory thalamus, which innervates L4 and L5 and represents a sensory percept that functions as the target for L2/3. It is also possible to extrapolate this model to higher cortical areas. Ascending cortico-cortical

connections can innervate middle layers of the higher-order cortex, including L4 and L5/6[29,30], potentially replacing, or working in tandem with, thalamic inputs as described in our study. Consequently, as we move up the cortical hierarchy the target for learning is no longer external sensory input, but rather a lower-order prediction relayed by a separate input, such as the ascending projections described above[31]. For example, in higher-order regions like the prefrontal cortex, predictions are largely decoupled from ongoing sensory activity. Instead, superficial L2/3 may predict a mnemonic sequence[32] which could be compared to stored hippocampal memories —acting as a reference, or target— which are sent to L5 of PFC[33]. In this example, the hippocampus may generate sequential patterns of activity that act as an implicit target for sequences generated in the cortex. This is consistent with evidence showing the importance of hippocampal communication in learning and the role of hippocampus-to-PFC input in signaling trial outcome feedback[34]. It is therefore possible that the cortical hierarchy exists along a gradient whereby predictions are grounded in sensory (external) vs. internal models of the world as we move from lower-order to higher-order regions. Indeed, anatomically, regions thought to encode internal models in the brain, such as the cerebellum and hippocampus, are strongly connected with higher-order brain regions[35,36]. In such instances, plasticity cannot rely on the tight temporal coincidence generated by simultaneous innervation of L4 and L5 by ascending axons from thalamus[15], however temporal synchrony across inputs and brain regions may instead be generated by neuronal oscillations[34].

How is information then transmitted between brain regions within the cortical hierarchy? The classical assumption is that feed-forward information is sent from L2/3 while feedback emanates from L5. Our model builds on work showing that both motor cortex and cingulate cortex provide top-down inputs to sensory areas that play an important role in modulating sensory responses[1,31,37]. In both cases, L2/3 and L5 pyramidal neurons mediate the projections to primary sensory areas[38,39]. Similarly, neurons in L2/3 and L5 of primary sensory areas also project to higher-order areas[38,40]. In some cases, such as sensory-motor connections, there may be no strict hierarchical organisation, and S1 and M1 may reciprocally share predictions and error signals related to both forwards and inverse models via parallel connections involving L2/3 and L5[41]. There is also evidence for non-canonical feedback circuits such as L4 inputs which give feedback to both deep and superficial layers[29].

These intracortical pathways are mediated by intratelencephalic (IT) projection neurons that populate both superficial and deep layers of the neocortex. However, within L5 there is also a separate subpopulation of subcortical projection neurons, so called pyramidal tract (PT) neurons, which typically do not project intracortically but instead target subcortical structures such as higher-order thalamus[42]. Higher-order nuclei integrate information from across the cortex by sampling different L5 PT cell inputs[30] and have been shown to convey mismatches between self-generated and sensory-derived visual input[43], which helps amplify prediction error signals in superficial layers[44]. Interestingly, while L2/3 stimulation causes an inhibition of L5 IT cells, consistent with both our model Fig. 7 and in vivo data[37], it also causes activation of L5 PT cells[11]. Therefore, under conditions where a mismatch occurs within superficial layers, it may produce a corresponding activation of L5 PT cells which transmit this signal either up or down the cortical hierarchy via transthalamic pathways[45].

Relationship with successor representations

In Successor Representation (SR) models in reinforcement learning[46], the core idea is to represent states based on the future states that are expected to follow them. SR encodes the expected discounted sum of future states for a given current state, facilitating the generalization of value estimates and enabling transfer learning across tasks. SR and the form of self-supervised learning (SSL) we focus on leverage temporal information to predict future states, making them valuable for tasks involving sequential data learning. SR accomplishes this within a reinforcement learning framework, while SSL, though often used in unsupervised contexts, can enhance RL by facilitating improved state representations. Given the links that have been made between SR and neuroscience, for instance in hippocampal networks[47], it would be interesting to establish more formal links between SR, SSL and cortical layers.

Supplementary figures

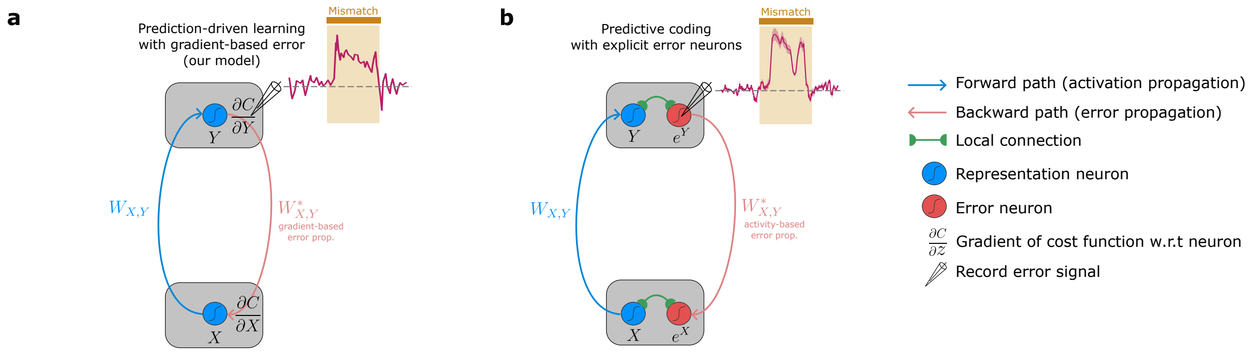

Figure S1. Comparison between our model and a predictive coding architecture. a, Our model implements error-driven learning similar to standard artificial neural networks, where errors are propagated directly through backward synapses to update neuronal activities. Therefore, all neurons in our model are both representation and error neurons (blue neuron with dashed red outline). **b,** In contrast, predictive coding explicitly represents prediction errors using dedicated error neurons (red) that are distinct from representation neurons (blue). Each representation neuron is paired with an error neuron that computes the mismatch between predictions and actual values.

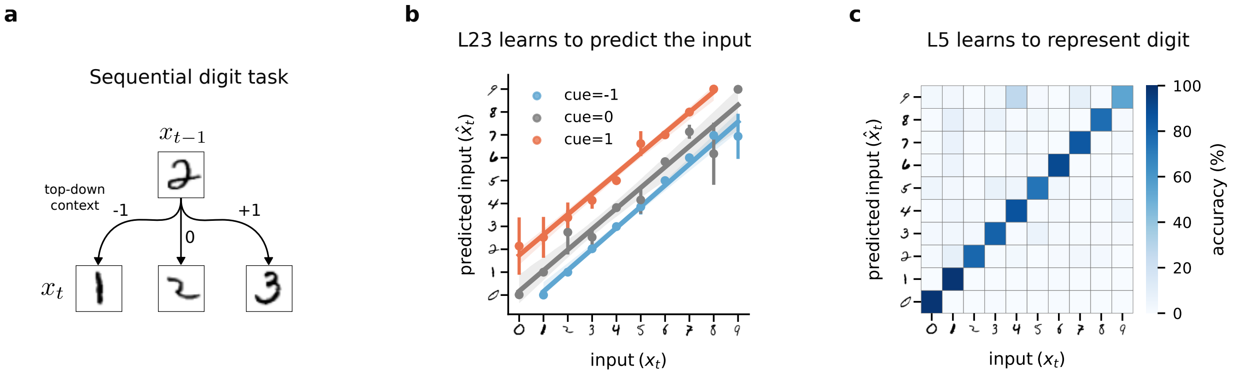

Figure S2. Learning a sequential digit task in a self-supervised canonical microcircuit. a, Sequential digit task used for training. The generative factor which is passed as top-down context determines the next digit. **b,** Prediction accuracy of a linear model trained on the output of L2/3. For a given input, L2/3 predicts the next possible input with high accuracy for all three cues (denoted by different colors). **c,** Confusion matrix showing the classification accuracy of a linear model trained on the output of L5. Error bars represent the standard error of the mean over 5 different initial conditions.

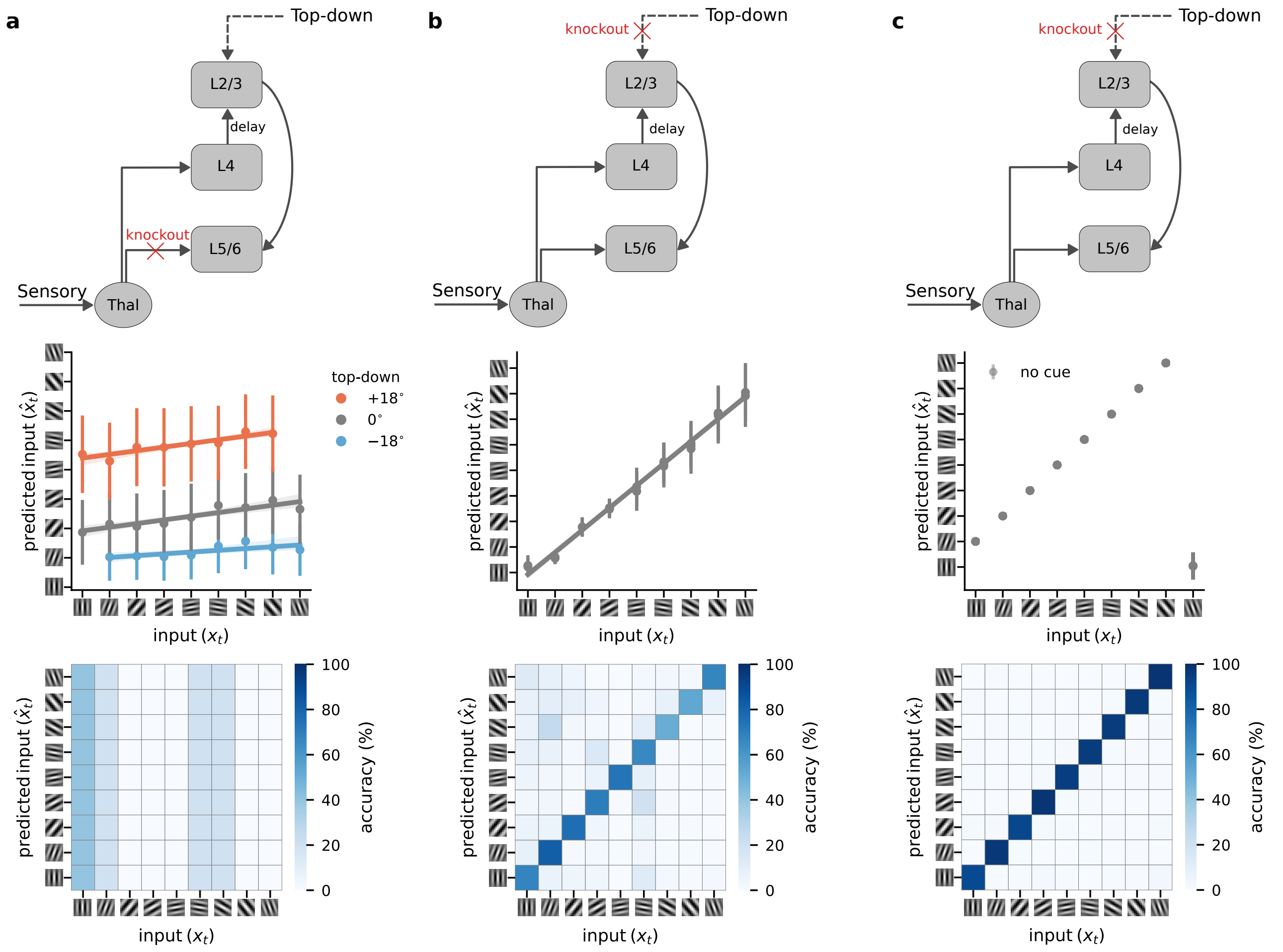

Figure S3. Prediction accuracy in L2/3 and L5 when connections are knocked-out. a, Connections from Thalamus to L5 are necessary for learning in both L2/3 and L5. **b,** Top-down input to L2/3 is crucial for L2/3 to predict the incoming input, while L5 performance is not significantly impacted when the top-down signal is deleted. **c,** Removal of top-down input to L2/3 in a deterministic task in which inputs always rotate clockwise (i.e. $+18°$), top-down input is not required for the task. In this case, removing the top-down does not have any effect on the task performance. Error bars represent the standard error of the mean over 5 different initial conditions.

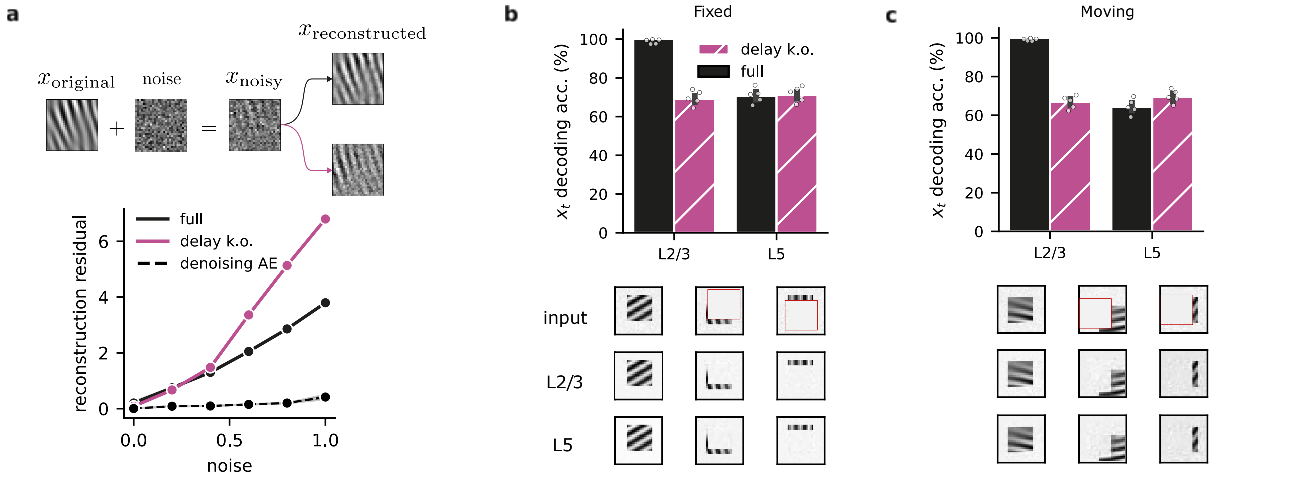

Figure S4. L4 to L2/3 delay increases denoising and robustness to occluded stimuli. a, L4 to L2/3 delay promotes noise suppression in L5 representations. Top: Schematic of noise added to the original inputs. Bottom: Noise-corrupted input samples lead to higher L5 reconstruction residuals ($\hat{\mathbf{x}}_t - \mathbf{x}_t$) when the L4-to-L2/3 delay is ablated (purple) compared to the full model (solid black). The dashed line represents the reconstruction residual for an autoencoder explicitly trained to denoise the input. **b,** Top: Decoding accuracy with and without L4 to L2/3 delay for a Gabor task with occlusion. Bottom: Three examples depicting L2/3's losing its ability to recover occluded information, similar to L5's incomplete reconstructions (top row: original occluded input; middle row: L2/3 reconstruction; bottom row: L5 reconstruction). **c,** Top: Accuracy with and without L4-to-L2/3 delay for a task in which Gabor patches move (top). Bottom: Examples further illustrate the robustness with moving Gabor patches (top row: original input with motion; middle row: L2/3 reconstruction; bottom row: L5 reconstruction). Error bars represent the standard error of the mean over 5 different initial conditions.

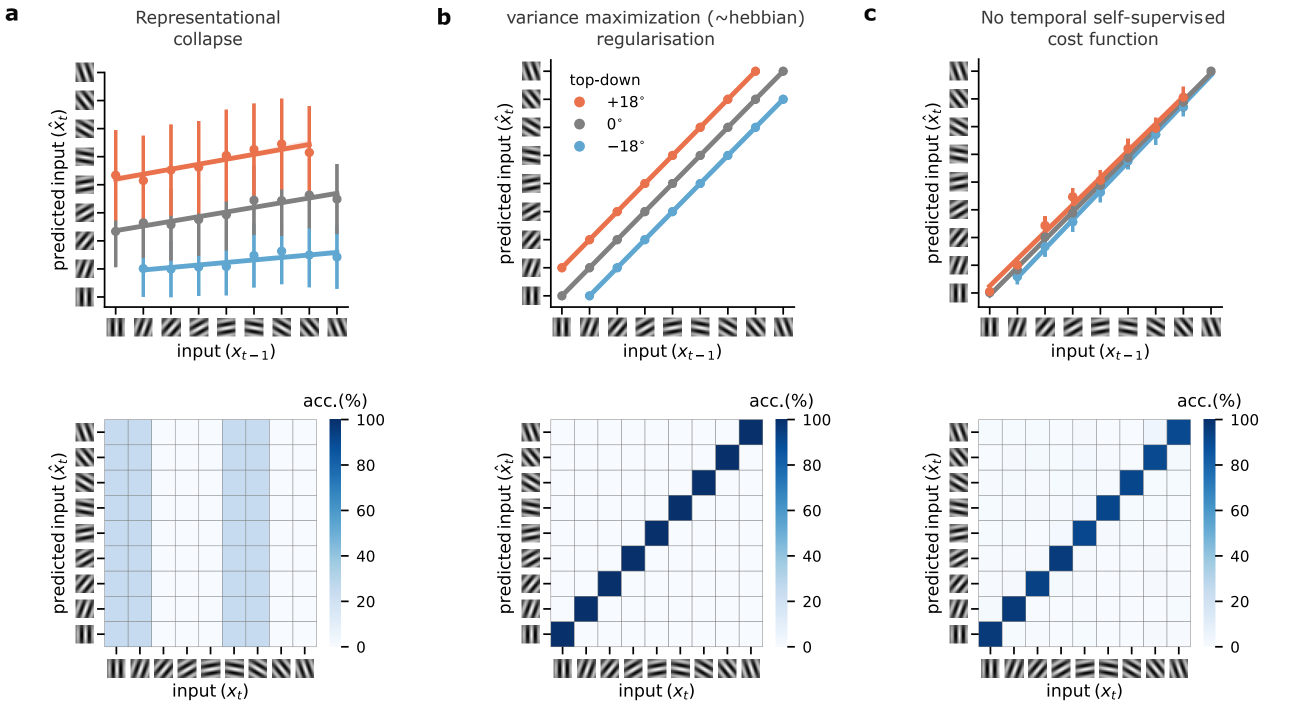

Figure S5. Regularisation of L5 and temporal self-supervision of L2/3 are crucial for learning. a, Removing the reconstruction loss from the L5 objective leads to representational collapse, as both L2/3 and L5 fail to learn meaningful representations. **b,** Replacing the L5 reconstruction loss with a variance maximization regulariser[3], analogous to Hebbian plasticity[4], enables both L2/3 and L5 to learn successfully. **c,** Replacing the L2/3 temporal self-supervised loss with a regression loss for predicting speed disrupts L2/3's ability to predict its input, even though L5 still learns to represent the data. Error bars represent the standard error of the mean over 5 different initial conditions.

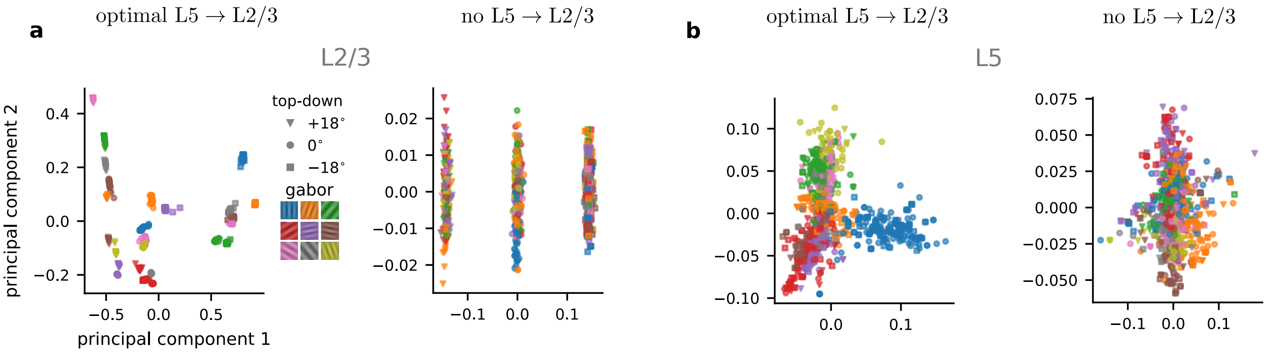

Figure S6. Low dimensional representations with optimal L5-to-L2/3 feedback in the Gabor temporal task. a, L2/3 representations are separated by context and Gabor orientation for optimal feedback (left) but are only grouped by context when the feedback is absent entirely (right). **b,** L5 representations between optimal feedback (left) and no feedback (right).

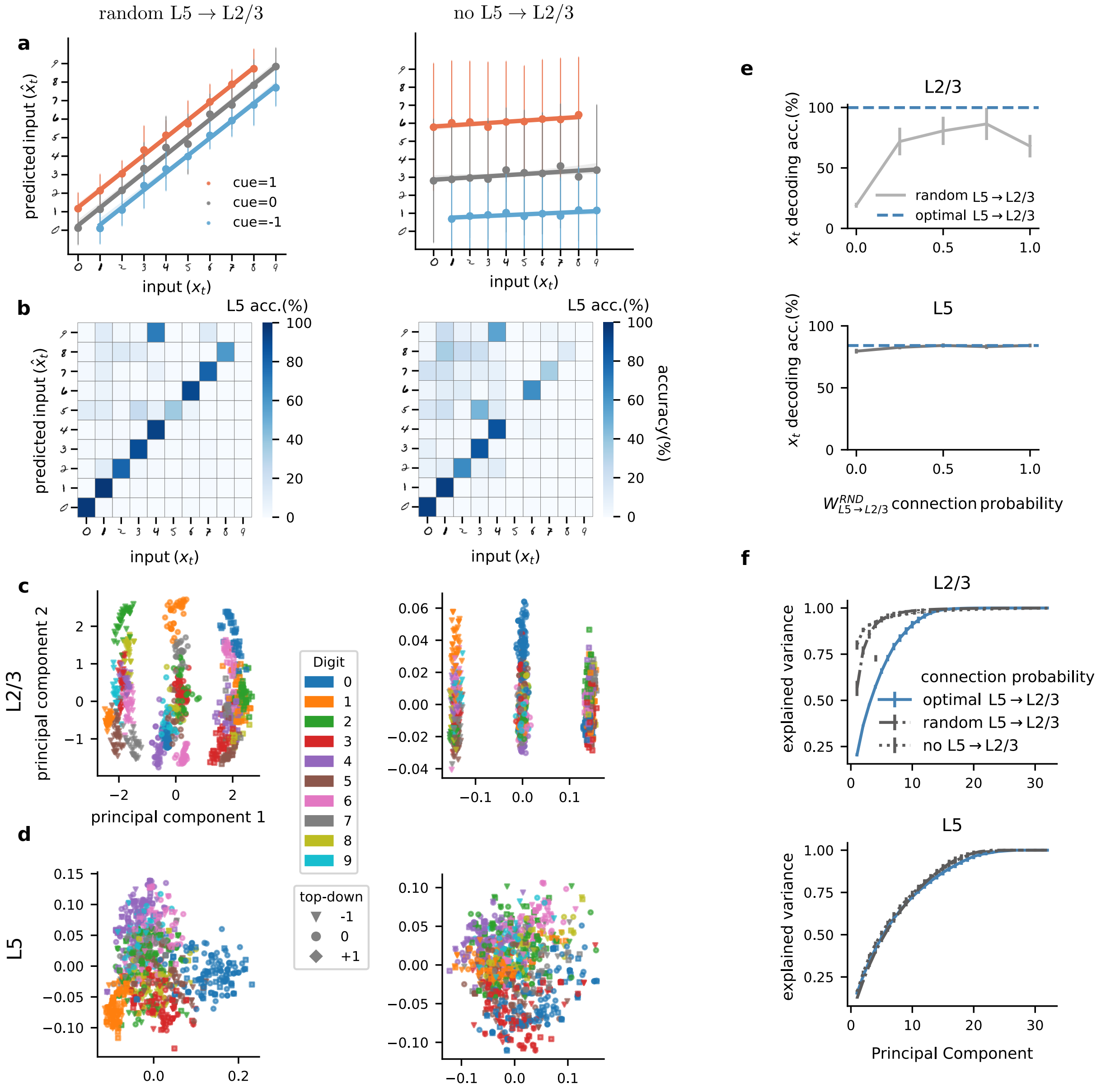

Figure S7. Role of feedback connections from L5 to L2/3 in learning a sequential digit task. a, L2/3 learns to predict the input with random feedback (left) but fails without feedback (right). **b,** L5 learns a good representation of the task with random feedback (left) and without feedback (right). **c,** L2/3 representations are separated by context and digit class for random feedback (left) but are only grouped by context when the feedback is absent entirely (right). **d,** L5 representation does not show a significant difference between random feedback (left) and no feedback (right). **e,** L2/3 linear prediction accuracy drops to chance level when the feedback is removed (top) while L5 classification accuracy is not impacted (bottom). **f,** The representation in L2/3 is mostly explained by the first few PCs for the 'no feedback' condition (top) while more PCs are required to explain the variance for the case with random feedback. L5 PCs explained variance is very similar in both conditions (bottom).

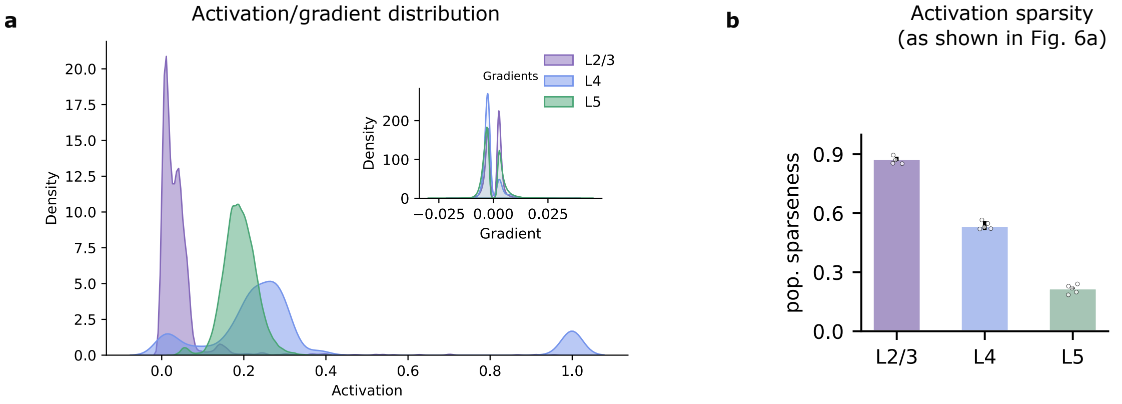

Figure S8. Distribution of neural activity and sparsity across cortical layers. a, Kernel density estimate (KDE) plots showing the activation distributions of neurons in layers L2/3, L4, and L5. The inset displays the gradients (errors) with respect to neurons in each layer, illustrating their distribution across the network. **b**, Population sparsity of activations across layers, replicating the sparsity plot from Figure 6, panel (a) of the main paper. The bar plot quantifies the degree of sparsity in each layer, showing that L2/3 exhibits the highest activation sparsity, followed by L4 and L5 (cf. panel a). Error bars represent the standard error of the mean over 5 different initial conditions. Error bars represent the standard error of the mean over 5 different initial conditions.

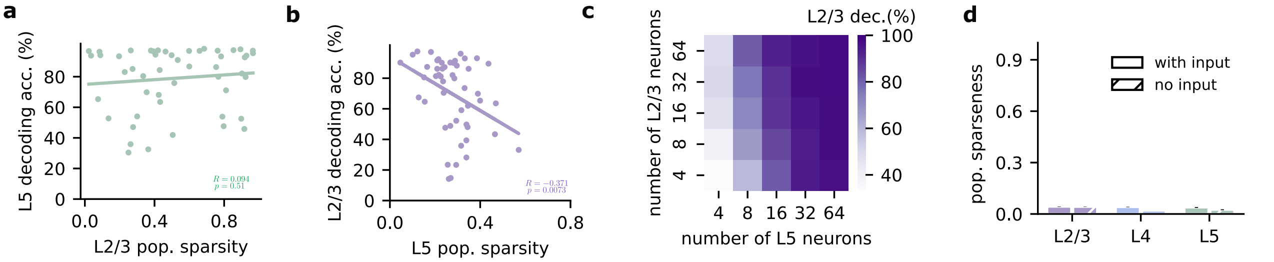

Figure S9. Sparsity and decoding accuracy of the current input in layer 2/3 and layer 5. a, L5 decoding accuracy as a function of L2/3 population sparsity. **b,** L2/3 decoding accuracy as a function of L5 population sparsity. Statistical tests are two-sided and no adjustments were made for multiple comparisons. **c,** L5 decoding accuracy as a function of different numbers of neurons in L2/3 and L5. **d,** Effect of input removal on the sparsity of neocortical layers before training. Error bars represent the standard error of the mean over 5 different initial conditions.

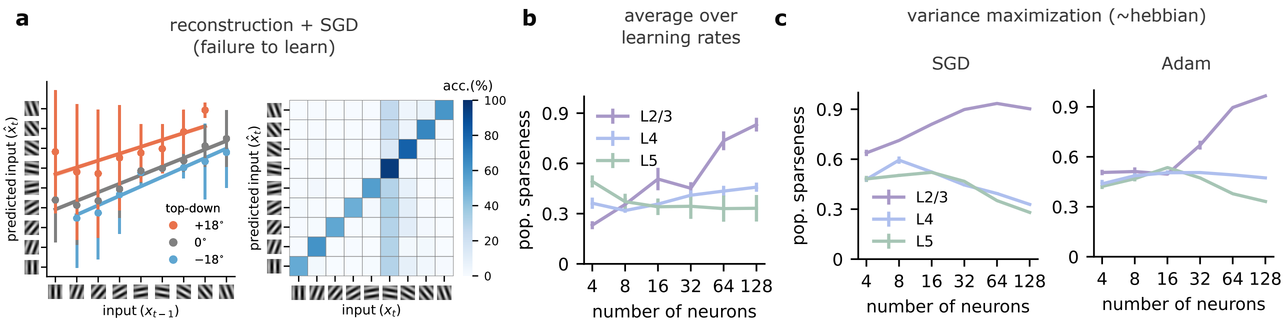

Figure S10. Influence of optimiser and regularisation on learning and sparsity. a, Regularisation of L5 loss results in learning failure, as the model gets stuck in local minima. The accuracy matrix highlights the poor performance. **b,** Average population sparseness across various learning rates [0.005, 0.001, 0.0005, 0.0001]. **c,** Replacing the reconstruction loss with variance maximization in L5 enables successful learning and produces consistent sparsity patterns across layers for both SGD (left) and Adam (right) optimisers. These results are consistent with the sparsity trends observed in Figure 6. Error bars represent the standard error of the mean over 5 different initial conditions.

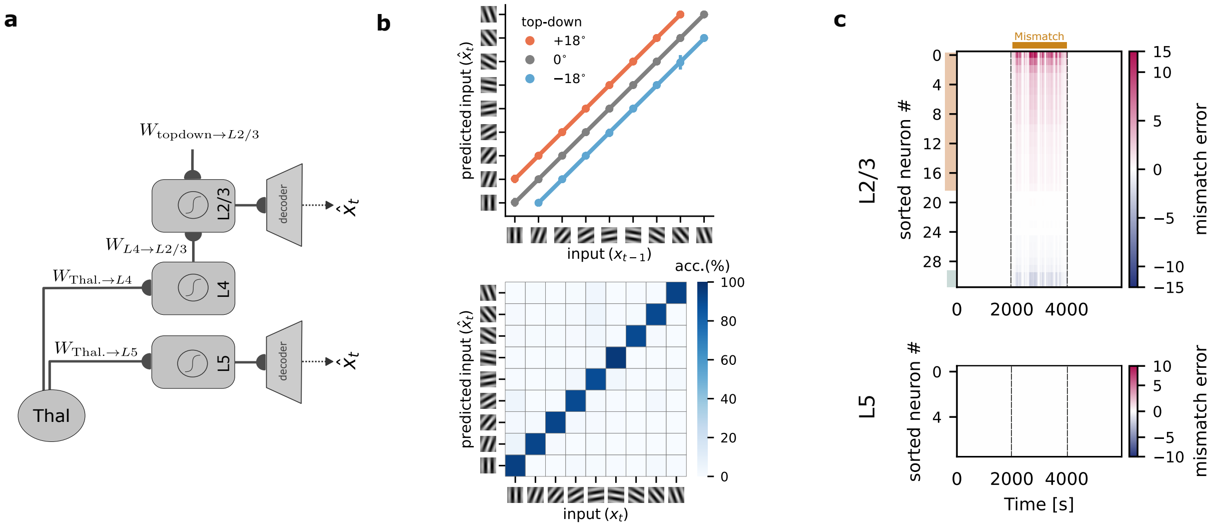

Figure S11. Separate self-supervised loss for L2/3 facilitates temporal learning but fails to capture L5 mismatch responses. **a,** Schematic of the model architecture incorporating a dedicated temporal self-supervised learning loss for layer 2/3 (L2/3). **b,** Demonstration of successful temporal learning in both L2/3 and L5, as evidenced by accurate prediction of the subsequent input. **c,** Comparison of L5 activity with experimental data from Jordan and Keller [1], revealing a discrepancy in mismatch responses. The model's L5 fails to exhibit the characteristic suppressed activity observed during mismatch periods, suggesting limitations of input-space reconstruction as a proxy for perceptual learning, consistent with observations in Balestriero and LeCun [6].

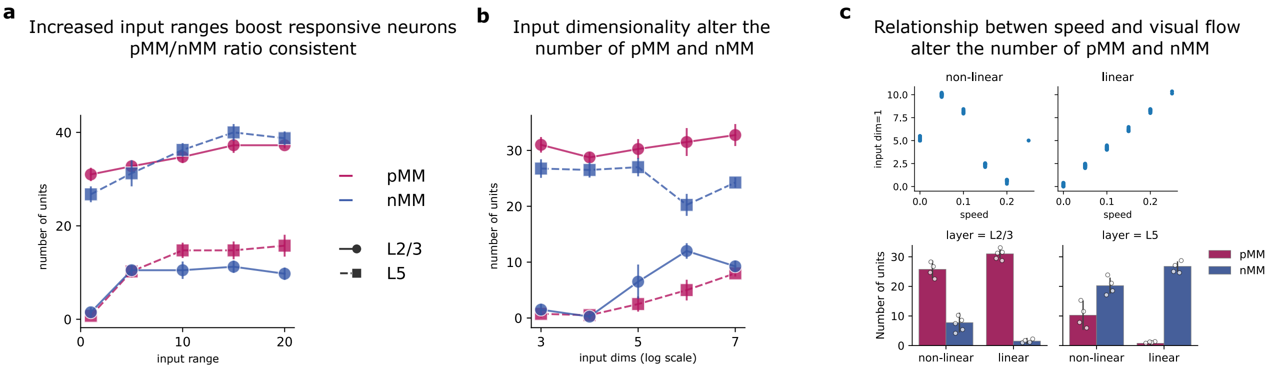

Figure S12. Mismatch errors across different model parameters. **a,** L2/3 and L5 prediction errors remain consistent across varying input values (pMM: positive mismatch errors; nMM: negative mismatch errors). **b,** L2/3 and L5 mismatch errors are robust to the dimension of the input. **c,** L2/3 and L5 mismatch errors persist with both linear and non-linear visual flow relationships to speed. Error bars represent the standard error of the mean over 5 different initial conditions.

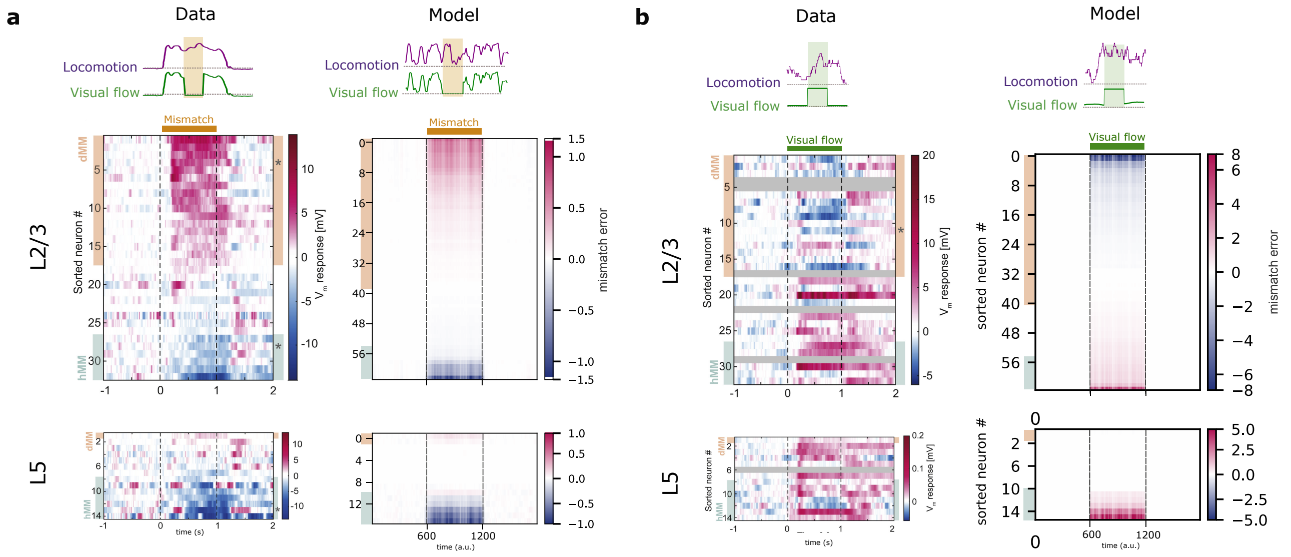

Figure S13. Model replicates reversed mismatch responses in open-loop conditions. a, Mismatch responses in L2/3 and L5 when visual input is halted during a sensorimotor task (same as in Fig. 7). **b,** Top: Schematic of the open-loop visual flow paradigm. Visual flow is initiated after a period of no stimulus (shaded green area). Bottom: Mismatch responses in L2/3 and L5. Neurons are sorted based on their responses in the visual flow halting experiment (Fig. 7). Experimental data (left) demonstrates a reversal of mismatch response polarity compared to the closed-loop halting experiment (cf. panel a). Model (right) accurately captures the flipped sign of mismatch responses, indicating anti-correlation with visual flow onset. Panels (a,b) were partially reprinted from Opposing Influence of Top-down and Bottom-up Input on Excitatory Layer 2/3 Neurons in Mouse Primary Visual Cortex, 108/6, Jordan R. and Keller G., Neuron, 1194-1206, Copyright (2020), with permission from Elsevier. Statistical tests are two-sided and no adjustments were made for multiple comparisons. Color bars indicate mismatch error magnitude.

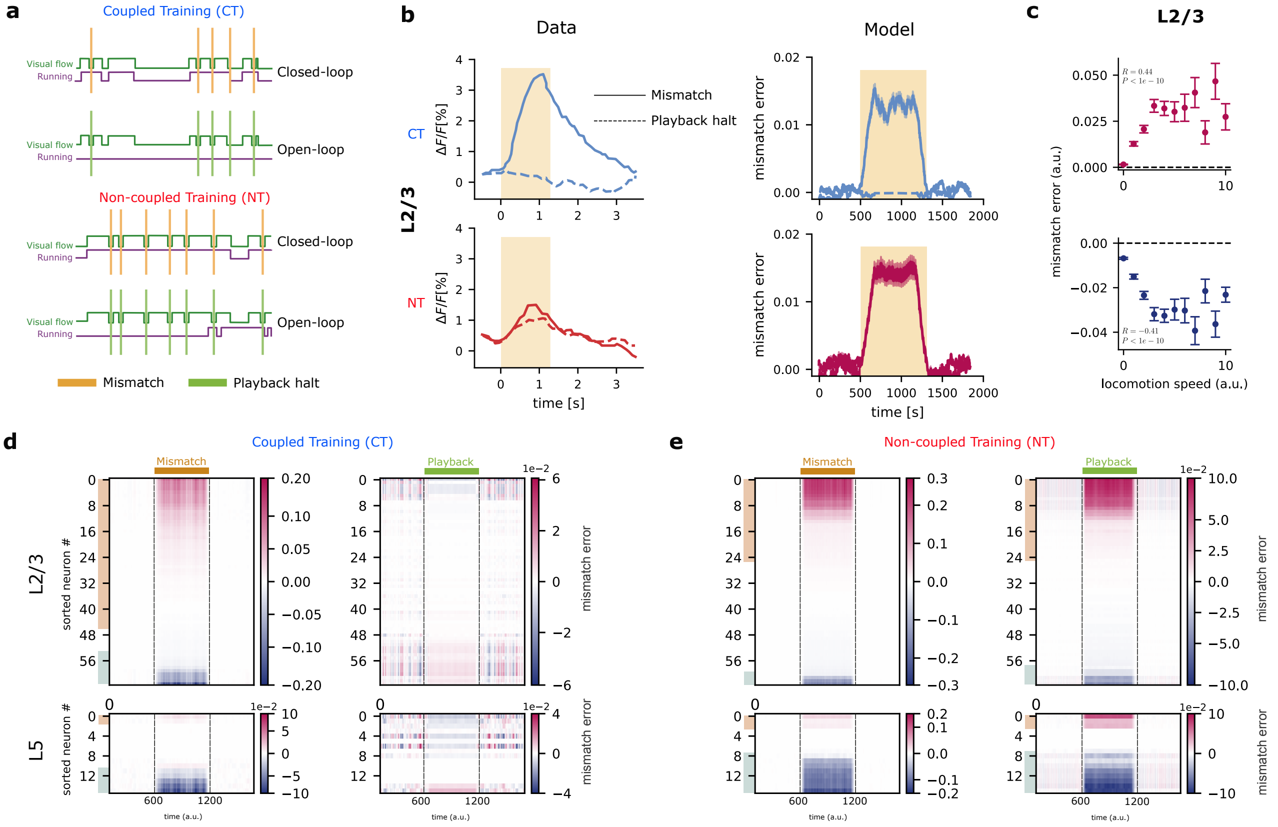

Figure S14. Recurrent model captures distinct neural responses to visuomotor mismatch in coupled and non-coupled training conditions. a, Schematic of the coupled training (CT, blue) and non-coupled training (NT, red) paradigms. Visual flow (green) is coupled to locomotion (purple) in CT but not NT. **b,** L2/3 neural responses to mismatch (solid lines) and playback halt (dashed lines) events in CT (blue) and NT (red) conditions. Left: Experimental data. Right: Model predictions. Orange shading indicates mismatch duration. **c,** Correlation between mismatch error and locomotion speed in L2/3 during the mismatch period for the RNN model. **d,** Model's mismatch responses in L2/3 (top) and L5 (bottom) under CT conditions for both mismatch and playback halt events. **e,** Same as **d** but for NT conditions. Panel a was adapted from Visuomotor Coupling Shapes the Functional Development of Mouse Visual Cortex, 169 / 7, Attinger A., Wang B., Keller G. B., Cell, 1291-1302, Copyright (2017), with permission from Elsevier. Error bars represent the standard error of the mean over 5 different initial conditions.

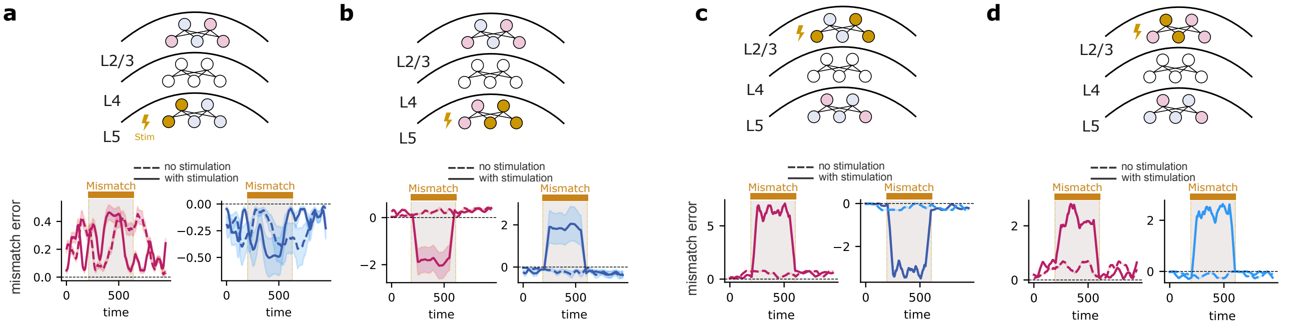

Figure S15. Mismatch errors in L2/3 and L5 after stimulating groups of neurons in L5 and L2/3, respectively. a, Increasing the activity of L5 neurons with positive errors during sensorimotor mismatch has a variable effect on mismatch errors in L2/3. **b,** Modulating the L5 neurons with negative errors inverts the errors in L2/3. **c,** Scaling the output of L2/3 neurons with positive error signals enhances the mismatch errors in L5. **d,** Modulation of L2/3 neurons with negative mismatch errors flips the sign of errors in L5. Error bars represent the standard error of the mean over 5 different initial conditions.

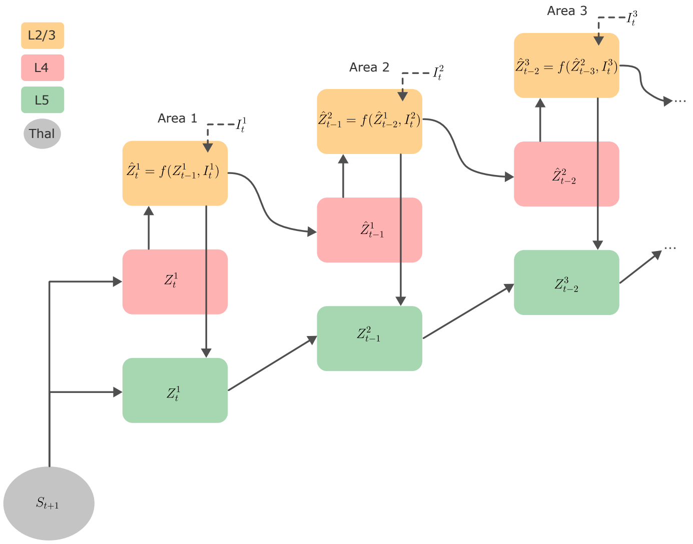

S_t Stimulus at timestep ***t***

I_t^n Top-down at timestep ***t*** to area ***n***

$\hat{Z}_t^n = f(X, Y)$ Prediction of target given X,Y

$Z_t^n = g(S)$ Stimulus representation at timestep ***t*** by area ***n***

Area	Target	Equations
1	Z_t^1	$Z_t^1 = g(S_t), \quad \hat{Z}_t^1 = f(Z_{t-1}^1, I_t^1)$
2	Z_{t-1}^2	$Z_{t-1}^2 = g(Z_{t-1}^1), \quad \hat{Z}_{t-1}^2 = f(\hat{Z}_{t-2}^1, I_t^2)$
3	Z_{t-2}^3	$Z_{t-2}^3 = g(Z_{t-2}^2), \quad \hat{Z}_{t-2}^3 = f(\hat{Z}_{t-3}^2, I_t^3)$
n	$Z_{t-(n-1)}^n$	$Z_{t-(n-1)}^n = g(Z_{t-(n-1)}^{n-1}), \quad \hat{Z}_{t-(n-1)}^n = f(\hat{Z}_{t-n}^{n-1}, I_t^n)$

Targets: representation by L5

$$Z_t^1 = g(S_{t-1})$$
$$Z_{t-1}^2 = g(Z_t^1) = g(g(S_{t-1}))$$
$$Z_{t-2}^3 = g(Z_{t-1}^2) = g(g(g(S_{t-2})))$$
$$Z_{t-(n-1)}^n = g^{(n)}(S_{t-(n-1)}) \quad \{n > 1\}$$

Predictions of the targets: output of L2/3

$$\hat{Z}_t^1 = f(g(S_{t-2}), I_t^1)$$
$$\hat{Z}_{t-1}^2 = f(f(g(S_{t-3}), I_{t-1}^1), I_t^2)$$
$$\hat{Z}_{t-2}^3 = f(f(f(g(S_{t-4}), I_{t-2}^1), I_{t-3}^2), I_t^3)$$
$$\hat{Z}_{t-(n-1)}^n = f^{n-1}(g(S_{t-n}), I_{t-n}^1, I_{t-n+1}^2, ..., I_t^n) \quad \{n > 1\}$$

Figure S16. Schematic of model across a cortical hierarchy. Schematic of the hierarchical organisation of cortical areas (left). In each column, L2/3 integrates prior input with top-down contextual information to generate a prediction in latent space, which is then compared with the activity in L5. The first cortical area predicts the current input using the previous input, while higher-order cortical area generate increasingly abstract predictions conditioned on predictions from the previous time step and extracting higher-level spatio-temporal features. Equations describing the flow of information within each cortical area (right), specifically at L2/3 and L5.

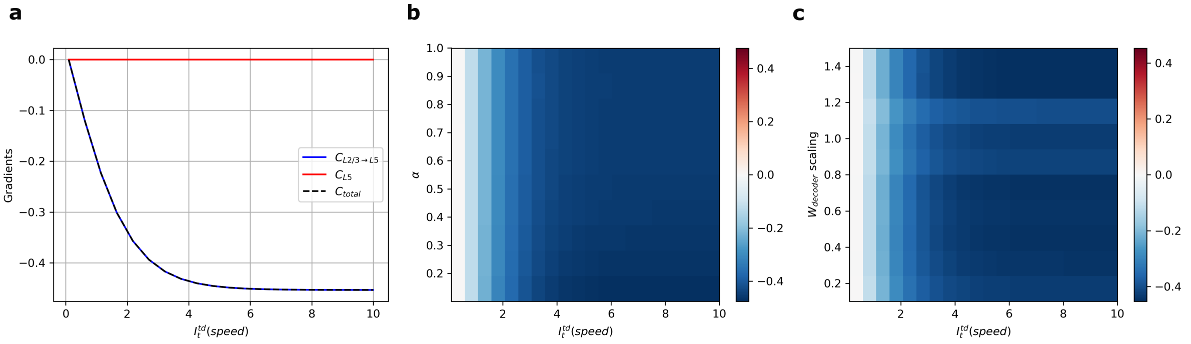

Figure S17. Gradient analysis of self-supervised and reconstruction costs during the mismatch period. a, The gradient of the total cost $\mathcal{C}_{\text{total}} = \lambda_p \mathcal{C}_{\text{L2/3}\rightarrow\text{L5}} + \lambda_r \mathcal{C}_{\text{L5}}$ with respect to L5 neurons, computed using the default parameters learned during model training. The total gradient is primarily driven by the self-supervised cost gradient $\mathcal{C}_{\text{L2/3}\rightarrow\text{L5}}$, while the gradient of the reconstruction cost $\mathcal{C}_{\text{L5}}$ remains close to zero. **b**, The effect of varying the modulation factor on the total cost gradient. The sign of the total gradient remains robust (negative) for different values of , as long as the top-down input is greater than one, a condition characteristic of the mismatch period. **c**, The impact of scaling W_{decoder} by a multiplicative factor. The total cost gradient with respect to L5 neurons remains robustly negative, indicating the stability of the mismatch-driven gradient dynamics.

References

[1] Rebecca Jordan and Georg B Keller. Opposing influence of top-down and bottom-up input on excitatory layer 2/3 neurons in mouse primary visual cortex. *Neuron* 108, 1194–1206 (2020).

[2] Timothy P Lillicrap, Daniel Cownden, Douglas B Tweed, and Colin J Akerman. Random synaptic feedback weights support error backpropagation for deep learning. *Nature communications* 7, 13276 (2016).

[3] Adrien Bardes, Jean Ponce, and Yann LeCun. Vicreg: Variance-invariance-covariance regularization for self-supervised learning. *arXiv preprint arXiv:2105.04906* (2021).

[4] M. S. Halvagal and F. Zenke. The combination of hebbian and predictive plasticity learns invariant object representations in deep sensory networks. *Nature Neuroscience* 26, 1906–1915 (2023).

[5] A. Attinger, B. Wang, and G. B. Keller. Visuomotor coupling shapes the functional development of mouse visual cortex. *Cell* 169, 1291–1302 (2017).

[6] R. Balestriero and Y. LeCun. Learning by reconstruction produces uninformative features for perception. *arXiv preprint arXiv:2402.11337* (2024).

[7] W. Greedy, H. W. Zhu, J. Pemberton, J. Mellor, and R. Ponte Costa. Single-phase deep learning in cortico-cortical networks. *Advances in Neural Information Processing Systems* 35, 24213–24225 (2022).

[8] A. Payeur, J. Guerguiev, F. Zenke, B. A. Richards, and R. Naud. Burst-dependent synaptic plasticity can coordinate learning in hierarchical circuits. *Nature neuroscience* 24, 1010–1019 (2021).

[9] Z. Friedenberger, E. Harkin, K. Tóth, and R. Naud. Silences, spikes and bursts: Three-part knot of the neural code. *The Journal of Physiology* 601, 5165–5193 (2023).

[10] J. Sacramento, R. Ponte Costa, Y. Bengio, and W. Senn. Dendritic cortical microcircuits approximate the backpropagation algorithm. *Advances in neural information processing systems* 31 (2018).

[11] T. Otsuka and Y. Kawaguchi. Pyramidal cell subtype-dependent cortical oscillatory activity regulates motor learning. *Communications Biology* 4, 495 (2021).

[12] S. R. Pluta, G. I. Telian, A. Naka, and H. Adesnik. Superficial layers suppress the deep layers to fine-tune cortical coding. *Journal of Neuroscience* 39, 2052–2064 (2019).

[13] J. C. Whittington and R. Bogacz. An approximation of the error backpropagation algorithm in a predictive coding network with local hebbian synaptic plasticity. *Neural computation* 29, 1229–1262 (2017).

[14] J. C. Whittington and R. Bogacz. Theories of error back-propagation in the brain. *Trends in cognitive sciences* 23, 235–250 (2019).

[15] C. M. Constantinople and R. M. Bruno. Deep cortical layers are activated directly by thalamus. *Science* 340, 1591–1594 (2013).

[16] J. H. Maunsell and J. R. Gibson. Visual response latencies in striate cortex of the macaque monkey. *journal of Neurophysiology* 68, 1332–1344 (1992).

[17] G. Plomp, C. M. Michel, and C. Quairiaux. Systematic population spike delays across cortical layers within and between primary sensory areas. *Scientific reports* 7, 1–14 (2017).

[18] W. B. Wilent and D. Contreras. Synaptic responses to whisker deflections in rat barrel cortex as a function of cortical layer and stimulus intensity. *Journal of Neuroscience* 24, 3985–3998 (2004).

[19] J. C. Wester and D. Contreras. Columnar interactions determine horizontal propagation of recurrent network activity in neocortex. *Journal of Neuroscience* 32, 5454–5471 (2012).

[20] K. Funayama, N. Hagura, H. Ban, and Y. Ikegaya. Functional organization of flash-induced v1 offline reactivation. *Journal of Neuroscience* 36, 11727–11738 (2016).

[21] G.-q. Bi and M.-m. Poo. Synaptic modifications in cultured hippocampal neurons: dependence on spike timing, synaptic strength, and postsynaptic cell type. *Journal of neuroscience* 18, 10464–10472 (1998).

[22] F. Gambino, S. Pagès, V. Kehayas, D. Baptista, R. Tatti, A. Carleton, and A. Holtmaat. Sensory-evoked ltp driven by dendritic plateau potentials in vivo. *Nature* 515, 116–119 (2014).

[23] K. C. Bittner, A. D. Milstein, C. Grienberger, S. Romani, and J. C. Magee. Behavioral time scale synaptic plasticity underlies ca1 place fields. *Science* 357, 1033–1036 (2017).

[24] R. Gao, R. L. Van den Brink, T. Pfeffer, and B. Voytek. Neuronal timescales are functionally dynamic and shaped by cortical microarchitecture. *Elife* 9, e61277 (2020).

[25] J. D. Murray, et al. A hierarchy of intrinsic timescales across primate cortex. *Nature neuroscience* 17, 1661–1663 (2014).

[26] Y. Kim, et al. Brain-wide maps reveal stereotyped cell-type-based cortical architecture and subcortical sexual dimorphism. *Cell* 171, 456–469 (2017).

[27] M. Stern, N. Istrate, and L. Mazzucato. A reservoir of timescales emerges in recurrent circuits with heterogeneous neural assemblies. *Elife* 12, e86552 (2023).

[28] X. Jiang, et al. Principles of connectivity among morphologically defined cell types in adult neocortex. *Science* 350, aac9462 (2015).

[29] G. Minamisawa, S. E. Kwon, M. Chevée, S. P. Brown, and D. H. O'Connor. A non-canonical feedback circuit for rapid interactions between somatosensory cortices. *Cell reports* 23, 2718–2731 (2018).

[30] J. A. Harris, et al. Hierarchical organization of cortical and thalamic connectivity. *Nature* 575, 195–202 (2019).

[31] G. B. Keller and T. D. Mrsic-Flogel. Predictive processing: a canonical cortical computation. *Neuron* 100, 424–435 (2018).

[32] Y. Xie, et al. Geometry of sequence working memory in macaque prefrontal cortex. *Science* 375, 632–639 (2022).

[33] P. G. Anastasiades and A. G. Carter. Circuit organization of the rodent medial prefrontal cortex. *Trends in neurosciences* 44, 550–563 (2021).

[34] S. L. Brincat and E. K. Miller. Frequency-specific hippocampal-prefrontal interactions during associative learning. *Nature neuroscience* 18, 576–581 (2015).

[35] F. A. Middleton and P. L. Strick. Anatomical evidence for cerebellar and basal ganglia involvement in higher cognitive function. *Science* 266, 458–461 (1994).

[36] S. Qiu, et al. Whole-brain spatial organization of hippocampal single-neuron projectomes. *Science* 383, eadj9198 (2024).

[37] G. B. Keller, T. Bonhoeffer, and M. Hübener. Sensorimotor mismatch signals in primary visual cortex of the behaving mouse. *Neuron* 74, 809–815 (2012).

[38] T. Mao, et al. Long-range neuronal circuits underlying the interaction between sensory and motor cortex. *Neuron* 72, 111–123 (2011).

[39] S. Zhang, et al. Long-range and local circuits for top-down modulation of visual cortex processing. *Science* 345, 660–665 (2014).

[40] E. J. Kim, et al. Extraction of distinct neuronal cell types from within a genetically continuous population. *Neuron* 107, 274–282 (2020).

[41] D. M. Wolpert, Z. Ghahramani, and M. I. Jordan. An internal model for sensorimotor integration. *Science* 269, 1880–1882 (1995).

[42] K. D. Harris and G. M. Shepherd. The neocortical circuit: themes and variations. *Nature neuroscience* 18, 170–181 (2015).

[43] M. M. Roth, et al. Thalamic nuclei convey diverse contextual information to layer 1 of visual cortex. *Nature neuroscience* 19, 299–307 (2016).

[44] S. Furutachi, A. D. Franklin, A. M. Aldea, T. D. Mrsic-Flogel, and S. B. Hofer. Cooperative thalamocortical circuit mechanism for sensory prediction errors. *Nature* 633, 1–9 (2024).

[45] S. M. Sherman. Thalamus plays a central role in ongoing cortical functioning. *Nature neuroscience* 19, 533–541 (2016).

[46] S. J. Gershman. The successor representation: its computational logic and neural substrates. *Journal of Neuroscience* 38, 7193–7200 (2018).

[47] K. L. Stachenfeld, M. M. Botvinick, and S. J. Gershman. The hippocampus as a predictive map. *Nature neuroscience* 20, 1643–1653 (2017).
