# Supplementary material for: Author Correction: Self-supervised predictive learning accounts for cortical layer-specificity
Source: Nat Commun. 2025 Oct 22;16:9354. doi: 10.1038/s41467-025-65076-5 (PMC12546837; doi:10.1038/s41467-025-65076-5)
Supplement: Supplementary file 1 — Original, uncorrected Fig. 2 [file 41467_2025_65076_MOESM1_ESM.pdf]

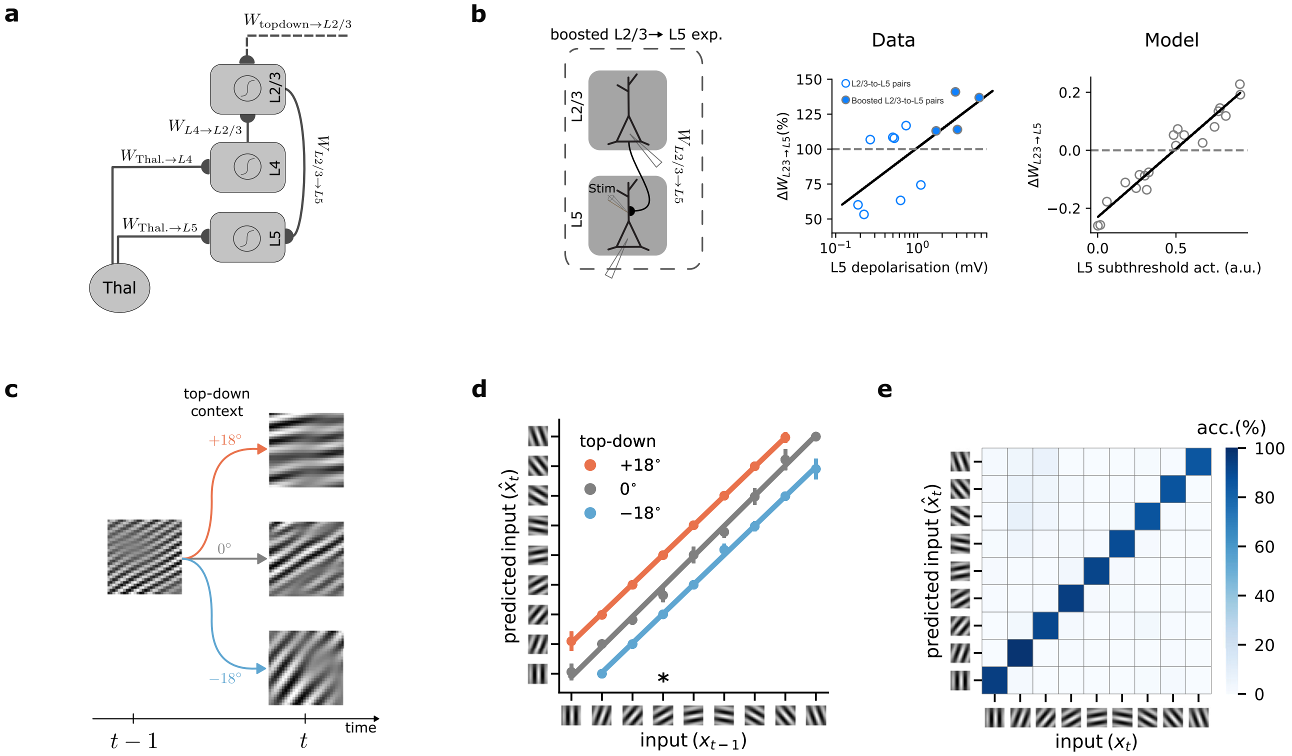

Fig. 2 | A model of temporal self-supervised learning in cortical circuits. **a** Schematic of the cortical circuit model. **b** Left: schematic of the experimental setup in which an extracellular electrode was used to boost L5 activity while inducing long-term synaptic plasticity on L2/3-to-L5 connections[28]. Middle: observed changes in synaptic weights as a function of L5 depolarization (scatter plot: individual data points, solid line: linear fit to the data). Right: L2/3-to-L5 learning rule as predicted by our model as a function of L5 activity for multiple randomly drawn samples of L2/3 and L5 activity (circles), and linear fit to the data points (solid line). **c**, Schematic of a sequential Gabor task. The generative factor provided to the model as top-down context at timestep t determines the orientation of the next Gabor patch at timestep $t+1$. **d** Decoding accuracy of a linear model trained on the output of L2/3. For a given input, L2/3 predicts the incoming sensory input with high accuracy. Colors represent the three possible conditions (−18°, 0°, and +18°). $*$ points to the example illustrated in (**b**). Error bars represent the standard error of the mean over five different initial conditions. **e** Confusion matrix for classification accuracy of a linear model trained on the output of L5. The matrix is calculated over five different initial conditions.
